# Pulsed Laser Fabrication of TiO_2_ Buffer Layers for Dye Sensitized Solar Cells

**DOI:** 10.3390/nano9050746

**Published:** 2019-05-15

**Authors:** Jeanina Lungu, Gabriel Socol, George E. Stan, Nicolaie Ştefan, Cătălin Luculescu, Adrian Georgescu, Gianina Popescu-Pelin, Gabriel Prodan, Mihai A. Gîrţu, Ion N. Mihăilescu

**Affiliations:** 1 Department of Physics, Ovidius University of Constanța, Constanța 900527, Romania; jmatei@univ-ovidius.ro (J.L.); contact.adriangeorgescu@gmail.com (A.G.); gprodan@univ-ovidius.ro (G.P.); 2 National Institute for Lasers, Plasma and Radiation Physics, P.O. Box MG-36, Măgurele 077125, Romania; gabriel.socol@inflpr.ro (G.S.); stefan.nicolaie@inflpr.ro (N.S.); catalin.luculescu@inflpr.ro (C.L.); gianina.popescu@inflpr.ro (G.P.-P.); 3 National Institute of Materials Physics, P.O. Box MG-7, Măgurele 077125, Romania; george_stan@infim.ro

**Keywords:** dye-sensitized solar cells, photovoltaic conversion efficiency, TiO_2_ thin films, pulsed laser deposition

## Abstract

We report on the fabrication of dye-sensitized solar cells with a TiO_2_ buffer layer between the transparent conductive oxide substrate and the mesoporous TiO_2_ film, in order to improve the photovoltaic conversion efficiency of the device. The buffer layer was fabricated by pulsed laser deposition whereas the mesoporous film by the doctor blade method, using TiO_2_ paste obtained by the sol–gel technique. The buffer layer was deposited in either oxygen (10 Pa and 50 Pa) or argon (10 Pa and 50 Pa) onto transparent conducting oxide glass kept at room temperature. The cross-section scanning electron microscopy image showed differences in layer morphology and thickness, depending on the deposition conditions. Transmission electron microscopy studies of the TiO_2_ buffer layers indicated that films consisted of grains with typical diameters of 10 nm to 30 nm. We found that the photovoltaic conversion efficiencies, determined under standard air mass 1.5 global (AM 1.5G) conditions, of the solar cells with a buffer layer are more than two times larger than those of the standard cells. The best performance was reached for buffer layers deposited at 10 Pa O_2_. We discuss the processes that take place in the device and emphasize the role of the brush-like buffer layer in the performance increase.

## 1. Introduction

Dye-sensitized solar cell technology continues to be a key technological domain as it allows for the production of low-cost energy from renewable sources [1], particularly under ambient lighting [2]. Dye-sensitized solar cells (DSSC) are photovoltaic devices consisting of a photoelectrode with a mesoporous layer of a nanocrystalline wide band gap semiconductor (such as anatase TiO_2_) on transparent conducting oxide, sensitized with a dye, and a counter electrode, for example platinized conductive glass, with a liquid or solid state electrolyte in-between [3,4]. The working principle of the device is based on light absorption in the dye, followed by transfer of the resulting photoelectrons from the excited level of the dye into the conduction band of TiO_2_. The electron diffuses via the semiconductor to the conducting glass substrate, passes through the external circuit and is carried by the redox electrolyte from the counter electrode back to the dye, to regenerate it [5].

For DSSCs with iodide/triiodide electrolyte, conversion efficiencies, in standard air mass 1.5 global (AM 1.5G) conditions, of more than 11% have been obtained using Ru(II)-polypyridyl complexes [6,7], which are widely used dyes in the photovoltaic devices [8,9]. More recently, by using porphyrin dyes, the efficiency reached 11.5% [10] and by replacing the iodine electrolyte with cobalt based complexes the efficiency went up to 12% [11]. Even higher performance was reported when using organic silyl-anchor dyes [12]. The use of perovskite light absorbers and organic hole conductors in a solid state cell resulted in efficiencies larger than 15% [13], which was subsequently further increased by design changes away from the DSSC structure to more than 22% [14].

Current studies dedicated to the mechanism of charge transport in DSSC have indicated that progress can be achieved through understanding and controlling the secondary processes inside cell. It has been found [15,16,17,18,19,20,21] that the cell performance is enhanced when employing an intermediate nanocrystalline layer of TiO_2_ between the transparent conducting glass substrate (FTO—fluorine-doped tin oxide) and the mesoporous TiO_2_ semiconductor. The buffer layer has the role to ensure a good mechanical contact, as well as to protect the electrodes against the dye solution action and the oxidation at high temperature and to reduce the recombination of electrons at the electrode/electrolyte interface.

A compact TiO_2_ layer on the conductive glass substrate can be prepared by different methods. Examples are electrochemical deposition [22,23], spray pyrolysis [24,25,26], screen printing [26,27,28], sol–gel [15,29,30], sputtering [31,32,33,34], chemical vapor deposition [35], atomic layer deposition [36,37,38], dip coating [39], spin coating [21,40], etc. It has been argued that the buffer layer prevents the back transfer of electrons from the conductive substrate to the electrolyte, leading to an increase in the DSSC conversion efficiency. For that reason the thin compact buffer film of TiO_2_ was also called a blocking layer. The range of the efficiency enhancement is wide, reports claiming 20% in the case of the sol–gel method [29], from 15–20% [32] up to 80% [34] for sputtering, almost 30% when mixing exfoliated titania nanosheets with anatase TiO_2_ nanoparticles [41].

In contrast with the numerous reports on TiO_2_ blocking layers obtained by the methods just mentioned, pulsed laser deposition (PLD) was less used for the preparation of buffer layers. An early report claimed about 4% efficiency increase when using pure TiO_2_ and more than 21% for Nb-doped TiO_2_ [42], whereas a later one indicated 42% efficiency enhancement [43].

In our study, we present the photovoltaic performance of DSSC devices fabricated with buffer layers obtained by PLD, to take advantage of the good adherence and the control of stoichiometry, crystallinity and purity of ablated materials. These benefits have attracted considerable interest during the last years for synthesizing high quality oxide thin films by PLD [44,45]. Moreover, the number and/or intensity of the laser pulses used for ablation, allow for the accurate control of the deposition rate [46], making it a unique method for obtaining oxide semiconductor nanostructures for DSSCs. The PLD TiO_2_ compact film was deposited onto FTO to generate a barrier between the conducting oxide and the mesoporous TiO_2_ layer prepared by the sol–gel method. We report on the characterization of the TiO_2_ buffer interlayers fabricated by PLD in either oxygen or argon on FTO glass substrates kept at room temperature. We also studied the influence of the buffer layer on the photoelectron conversion process and the performance of DSSCs.

## 2. Materials and Methods

### 2.1. Solar Cell Fabrication

Both electrodes were obtained starting from transparent conductive glass substrates, which consisted of soda lime glass sheets of 2.2 mm thickness, covered with a conductive layer of fluorine-doped tin oxide (SnO_2_:F; FTO) with a 7 Ω/square resistivity (available from Solaronix). Before the preparation of the electrodes, the conductive glass was ultrasonically cleaned for 15 min in acetone, ethanol and deionized water, respectively, to remove any impurities, and then blown dry with high purity nitrogen. The first step in the preparation of the photoelectrode was the ablation of the pure TiO_2_ target on clean FTO glasses, using an excimer laser source KrF* (λ = 248 nm, τ_FWHM_ = 25 ns). The thin films deposition was performed inside a stainless steel irradiation chamber at room temperature. The target was produced from homogeneous anatase TiO_2_ powder (Sigma-Aldrich Corp., St. Louis, MO, USA, 637,254, 99.7% purity) with nanoparticle sizes of less than 25 nm mixed in agate mortar grinder. The ground TiO_2_ powder was initially pressed at 5 MPa and after that sintered for 6 h at 1100 °C in air, with a heating/cooling ratio of 20 °C/min, to obtain compact pellets. The laser beam incidence angle onto the target surface was about 45° and the target-substrate separation distance was set at 4 cm. For the deposition of one film 3 × 10^3^ subsequent laser pulses were applied, succeeding to each other with a repetition rate of 2 Hz. The targets were irradiated with a laser fluence of 2 J/cm^2^.

The buffer layers were obtained at 10 Pa and 50 Pa, by circulating high purity (99.999%) oxygen or argon inside the irradiation chamber, with the aid of a calibrated inlet. The dynamic pressure was monitored with an MKS 100 controller. The samples were labeled TO and TAR for layers deposited in oxygen or argon, respectively, and with two extra digits indicating the pressure, in Pa.

The electrodes obtained with a buffer layer under oxygen and argon atmosphere were used to further fabricate DSSC devices. The active layer was fabricated using a TiO_2_ paste prepared by the Pechini type sol–gel method [47], starting from a polyester-based titanium sol. The sol contained a mixture of precursor with molar ratio of 1:4:16 [Ti(iOPr)_4_:citric acid:ethylene glycol]. The paste was obtained by grinding in a mortar the nanocrystalline anatase TiO_2_ powder (Sigma-Aldrich Corp., St. Louis, MO, USA, 637,254). The sol–gel solution had 7:1 molar ratio between TiO_2_ and titanium (IV) isopropoxide [Ti(iOPr)_4_] (Sigma-Aldrich Corp., St. Louis, MO, USA) [48,49]. The paste was spread on the TiO_2_ buffer layer by the ‘doctor-blade’ technique. TiO_2_ films were annealed at 450 °C for 1 h in air and left until cooling to room temperature [18].

The last step in the preparation of the photoelectrodes was the sensitization of the mesoporous film of nanocrystalline TiO_2_ grains with the N719 (Ruthenium 535-bisTBA) dye, cis-diisothiocyanato-bis(2,20-bipyridyl-4,40-carboxylato) ruthenium(II) bis (tetrabutylammonium) [50] (from Solaronix S.A., Aubonne, Switzerland). The photoelectrodes were immersed in the dye solution (0.2 mM in absolute ethanol) at a temperature of 80 °C for 2 h, then rinsed with absolute ethanol and dried in the oven at 80 °C for 10 min.

The counter electrodes were obtained by spreading a few drops of Platisol T (Solaronix) onto the FTO and drying at 450 °C for 10 min. Both types of electrodes were stored in desiccators before use. The DSSCs were assembled by pressing the photoelectrode against the counterelectrode with small bulldog clips [51,52]. Finally, the electrolyte (Iodolyte Z-50, from Solaronix S.A., Aubonne, Switzerland) was injected between the electrodes filling up the space by capillary action.

### 2.2. Measurements

X-ray diffraction (XRD) was used to assess the structural properties of the samples and identify the crystalline phases. The XRD measurements were performed in Bragg-Brentano geometry with a D8 Advance diffractometer (Bruker Corp., Billerica, MA, USA), equipped with CuKα (λ = 1.5418 Å) radiation and a high efficiency one-dimensional LynxEye™ detector operated in integration mode. The patterns were recorded in the 2θ range 20°–60°, using a step size of 0.04° and a time per step of 5 s.

The surface morphology of samples was investigated by scanning electron microscopy (SEM) using a Inspect S electron microscope (FEI Co., Hillsboro, OR, USA). The SEM measurements were performed in high vacuum, at 20 kV acceleration voltage, using the secondary electrons acquisition mode. Before the SEM examination, a thin Au film was applied to coat the samples to prevent the electrical charging. The film uniformity and thickness were estimated based on cross-section SEM micrographs. The chemical analyses of the TiO_2_ films were carried out by means of energy dispersive spectroscopy (EDS). Additionally, transmission electron microscopy (TEM) examinations were conducted using a *CM 120 ST* microscope (Philips N.V., Amsterdam, The Netherlands), which operated at 120 kV and had a point-to-point resolution of 0.24 nm. The samples for the TEM investigation were dispersed in ethylic alcohol and collected on 300 mesh coated grids [53,54].

The optical transmission properties were analyzed with a Cintra 10e UV-Vis spectrophotometer (GBC Scientific Equipment Pty Ltd., Braeside VIC, Australia), in the range 300–1200 nm. The electro-optic parameters of the devices, particularly the fill factor (*FF*), the photovoltaic conversion efficiency (*η*), the short circuit current (*I_SC_*) and the open circuit voltage (*V_OC_*), were measured using a home-made small area solar simulator [55], which provided AM 1.5G standard irradiation conditions. The solar simulator illuminated the surface of the DSSCs through a circular slit of 10 mm diameter, such that the area exposed to light was of about 0.785 cm^2^. The measurement of current and voltage was performed using two digital *MS8050* multimeters (Mastech Group International Ltd., Hong Kong, China) and a precision decade resistance box. The measurements were carried out by changing the load resistance, at intervals of about 45 s, which allowed enough time for stable reading.

## 3. Results

At visual inspection, PLD TiO_2_ films were uniform, and adherent (even at edges). TO10 and TAR10 films were compact, completely transparent, with rainbow reflection in daylight. TO50 films were faintly translucent, exhibiting a surface with porous aspect, whilst TAR50 films were slightly smoky, but compact.

**Figure 1:**
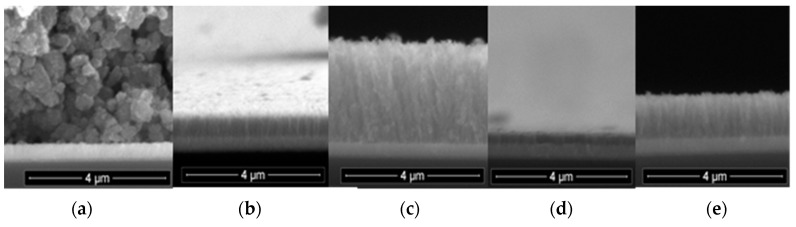
SEM micrographs of samples with the mesoporous TiO_2_ deposited directly on fluorine-doped tin oxide (FTO) (**a**) and structures with TiO_2_ buffer layers deposited by pulsed laser deposition (PLD) on FTO in oxygen at 10 Pa (TO10) (**b**), and 50 Pa (TO50) (**c**), in argon at 10 Pa (TAR10) (**d**), and at 50 Pa (TAR50) (**e**).

Top-view SEM investigations of the TiO_2_ layer deposited on FTO (Figure 1a) revealed nanoparticles with homogeneous shape and size dimension, in agreement with previous studies [56,57,58]. The buffer-layers display a brush-like compact structure consisting of TiO_2_ nanobars (Figure 1). The cross-section SEM images allowed the estimation of the layer thickness, as shown in Table 1. The samples obtained by low pressure PLD are systematically thinner than those deposited under higher pressure. One possible explanation may be that at higher pressure, due to multiple collisions with atoms/molecules of the gas, the ablated species lose more of their kinetic energy, resulting in a more confined plasma plume and the growth of larger nanobars.

**Figure 2:**
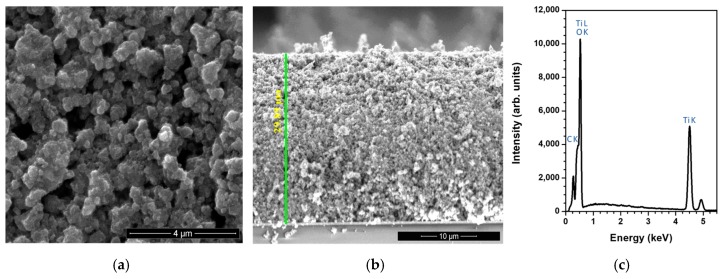
SEM micrographs, top-view (**a**) and cross-section showing a thickness of 29.89 μm (**b**), and energy dispersive spectroscopy (EDS) spectrum (**c**) of the TiO_2_ mesoporous layer.

The top-view and cross-view SEM images of the deposited TiO_2_ layer pointed to a mesoporous morphology (Figure 2). The EDS spectrum demonstrated that no additional elements were present in the mesoporous layer, except for carbon, which often contaminates the surface of samples kept in air. Carbon was not taken into account in the quantification of the atomic number, absorption and fluorescence (ZAF) corrections.

**Figure 3:**
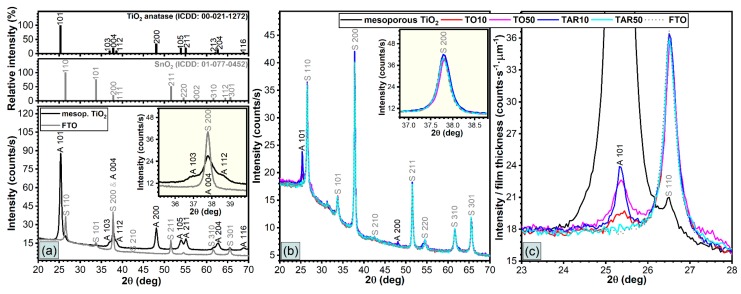
XRD patterns of the TiO_2_ mesoporous film and of the TiO_2_ buffer layers deposited by PLD on FTO glass substrates: TiO_2_ mesoporous film vs. FTO substrate (**a**); PLD layers vs. FTO substrate (**b**) and detail of the XRD pattern characteristic to the anatase (101) peak (**c**).

The XRD patterns of the mesoporous TiO_2_ film, and the buffer layers are given in Figure 3. Except for the TAR50 sample, which seemed amorphous, all other layers suggested a monophasic structure, exhibiting the maxima only of TiO_2_-anatase (ICDD: 00-021-1272). This phase is characteristic to PLD films annealed at 450 °C [23], along with the peaks of the electrode substrate layer (SnO_2_, ICDD:01-077-0452), which covers the glass substrates. No texturing of the anatase films was noticed. However, in the case of the FTO layer, a clear texturing in the (200) crystalline direction was evident. This FTO peak was superimposing, in the case of the mesoporous film, or even obscuring the (004) line of anatase, in the buffer layer samples.

In the case of the TAR50 sample no TiO_2_ peaks were evidenced, thus suggesting the amorphous status of these films. TAR50 samples were amorphous likely because at high pressure the velocity of the ablated species significantly decreased due to the collisions with “huge” Ar atoms. Accordingly, the energy necessary for the nucleation of crystals was significantly reduced in the case of these films.

A simple attenuation calculus for the anatase phase, at the Cu Ka energy, based upon a hypothetical density of ~4 g/cm^3^, indicated that the X-ray beam scattered at 2θ ≈ 25.3° would be totally attenuated by a dense film with a thickness in excess of 5 μm. The average crystal coherence length, as estimated via Scherrer equation, is collected in Table 2. The crystal coherence length of the anatase coatings was estimated from the full width at half maximum (FWHM) of the (101) diffraction line. The instrumental broadening of the diffraction line was corrected using a CeO_2_ highly crystalline laboratory control. We note that Scherrer equation assumes a negligible contribution of the lattice strain to the peak broadening.

**Figure 4:**
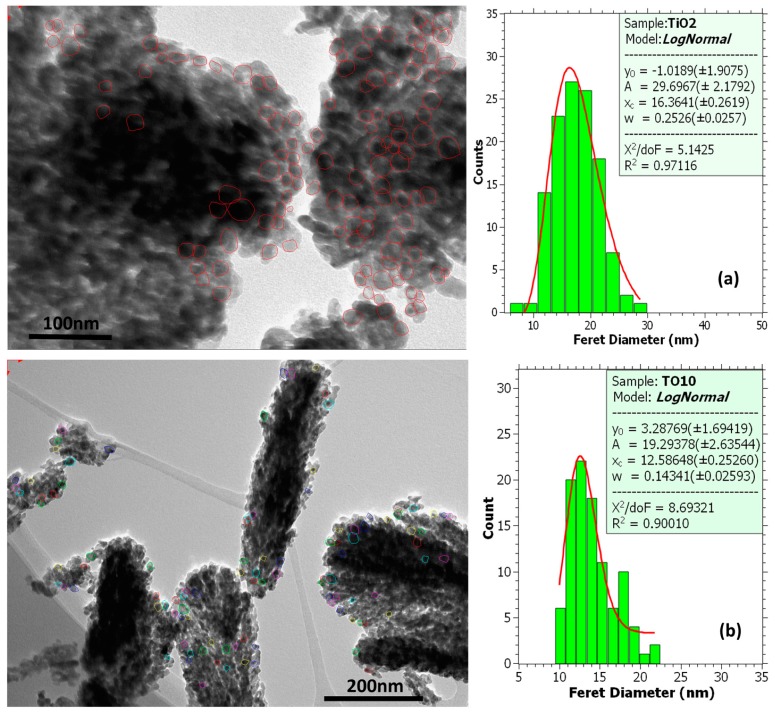

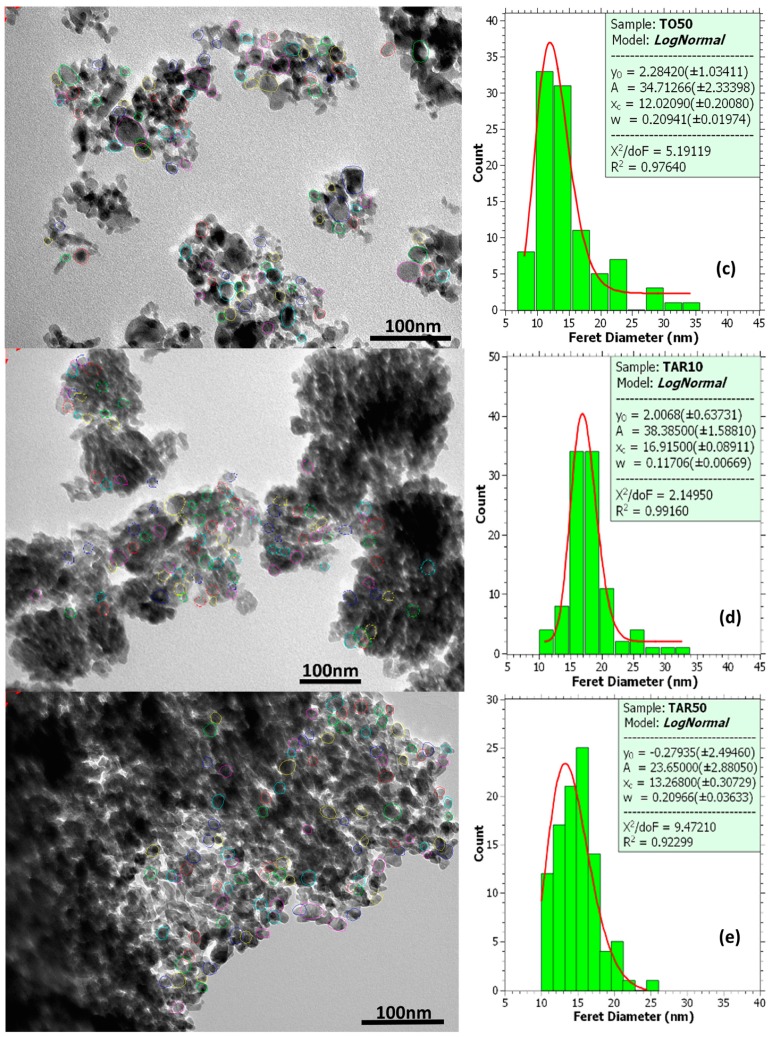
TEM image, selected area electron diffraction (SAED) pattern and distribution of grain size for the mesoporous TiO_2_ film (**a**), and the buffer layers: TO10 (**b**), TO50 (**c**), TAR10 (**d**) and TAR50 (**e**).

TEM images, along with grain size histograms (lognormal fitted), for mesoporous TiO_2_ films and PLD TiO_2_ buffer layers are shown in Figure 4. The TEM specimens were prepared by detaching fragments from the film onto the grid [31]. The grains were identified manually with the Olympus Soft Imaging Solutions GmbH, Münster, Germany iTEM TEM imaging platform. The grain sizes were distributed between 10 nm and 30 nm, with the average grain size, reported in Table 2, in the range of 12–17 nm. Although these values are smaller than those inferred by XRD studies, the different results obtained by the two methods are not incompatible. In the case of the XRD estimations the use of a single well-defined peak is a limitation, whereas in the case of the TEM assessments, the use of a particular sample area and of a finite number of grains influence the accuracy of the grain size distribution function. Within the limits of validity of the approximations used and the corresponding error bars of the two independent estimations, we suggest that, overall, the results were consistent.

**Figure 5:**
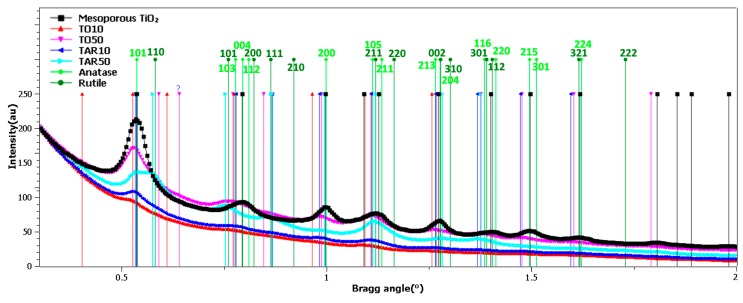
SAED profile obtained using CRISP2 software [59] with the ELD module [60] for the mesoporous TiO_2_ film and the buffer layers: TO10, TO50, TAR10 and TAR50. The indexing of anatase and rutile phases, which was performed according to Refs. [61,62], is represented as: Anatase = diamonds and rutile = circles.

Figure 5 presents the selected area electron diffraction (SAED) profiles extracted from the SAED patterns (see Supplementary Materials). For the mesoporous TiO_2_ film, Figure 5 reveals strong signature peaks of the anatase phase, particularly for the (101) plane. Other noticeable peaks were indicative of the (004) and (200) planes. In contrast, the TO10 and TAR10 samples showed much lower intensity peaks, which could still be associated with the anatase phase of TiO_2_, although the presence of the rutile phase cannot be excluded. Intermediate intensity peaks were present in the spectrum of TO50, pointing to the existence of wide crystallinity regions, with only small amorphous phase contributions. A clear mixture of anatase and rutile phases was noticeable in the TAR50 spectrum, particularly for the (110) plane of the rutile structure. For the TAR50 samples, the SAED and RDF data suggested the presence of crystalline regions along with the amorphous phase, in contrast to the XRD patterns. The differences may be caused by the irregularities of the samples, because TEM investigates a particular area of the sample whereas XRD provides a global perspective.

**Figure 6:**
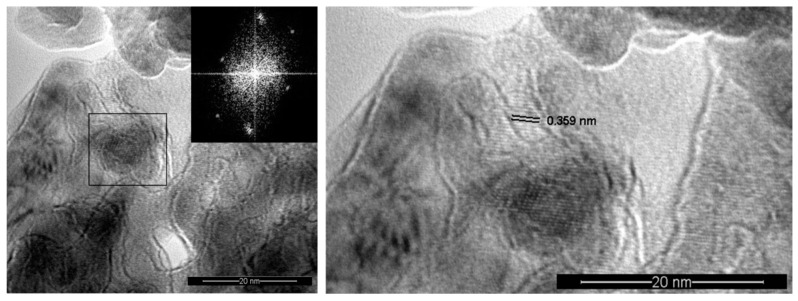
High resolution TEM image with inset of the corresponding fast Fourier transformer presentation of the selected area of the TiO_2_ buffer layer deposited at 10 Pa oxygen (TO10) (**left**) and a detail showing the interplanar distance corresponding to the anatase (101) plane (**right**).

Figure 6 displays a typical high-resolution transmission electron microscopy (HRTEM) image of a TO10 sample with an inset showing the fast Fourier transform (FFT) power spectrum of a specific sample area. By indexing the FFT spectrum one obtains interplanar distances of 0.359 nm assigned to the (101) lattice plane reflections of the tetragonal anatase TiO_2_ phase. The anatase phase nanocrystallites could be also identified from the 0.359 nm lattice fringes visible in the HRTEM image.

**Figure 7:**
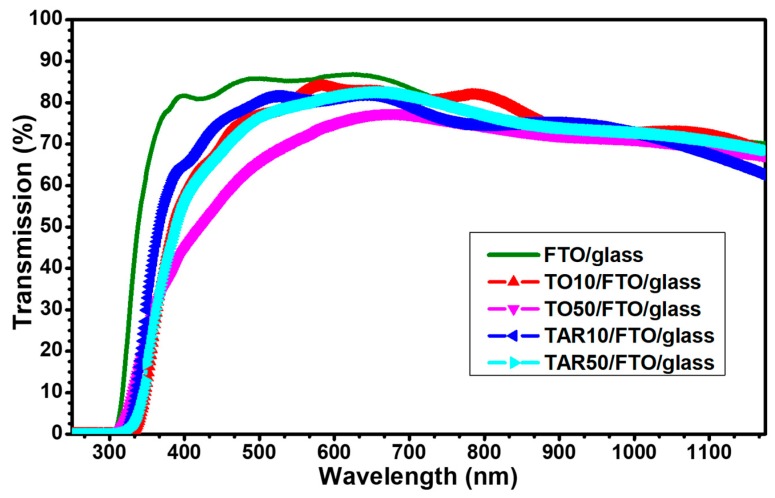
Transmission spectra of mesoporous TiO_2_ film and of TiO_2_ buffer layers deposited by PLD on FTO glass substrates.

The UV/Vis transmission spectra of the TiO_2_ buffer layers obtained by PLD on FTO glass substrate are presented in Figure 7. It can be seen that the average transmittance value, in the visible range, reached around 75–80%. The transmission of TO50 samples was slightly lower, likely due to their larger thickness.

**Figure 8:**
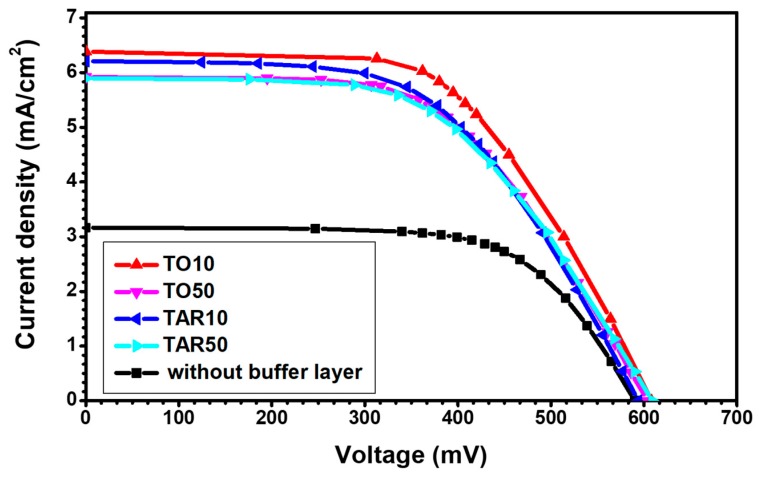
Current-voltage (*I-V*) curves for typical DSSCs fabricated using photo-electrodes with various buffer layers: TO10 (dashed line), TO50 (dotted line), TAR10 (dashed dotted line) or TAR50 (dashed dotted dashed line). The measurements were performed under standard air mass 1.5 global (AM 1.5G) illumination conditions.

Table 3 displays the typical values of the key parameters of the DSSCs fabricated using photo-electrodes with various PLD buffer layers, determined by electro-optical measurements. The *I-V* curves for a few selected cases are illustrated in Figure 8. For comparison, we provided values for the case of a DSSC without a buffer layer.

One remark is that the photovoltaic conversion efficiency for solar cells fabricated with the buffer layer was more than twice larger than the efficiency of the devices with the mesoporous TiO_2_ film applied directly to the FTO. As the open-circuit voltage was about the same, crucial in determining the significantly higher efficiency was the much larger short-circuit current density. By introducing a buffer layer we obtained a substantial increase of the short circuit current density, *J*_sc_, from 3.166 mA/cm^2^ for cells without buffer layer to 8.136 for TO10.

The fill factor was within the typical range of solar cells 0.5 to 0.8, although closer to the lower rather than the higher limit. As the highest value was reached for the device without a buffer layer, an analysis of the device losses is necessary.

## 4. Discussion

**Figure 9:**
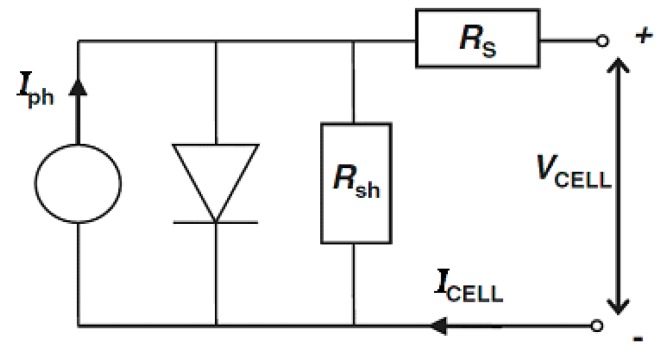
Equivalent circuit model of a solar cell.

The efficiency of solar cells was affected by the losses due to parallel (shunt) resistance (*R*_sh_) and series resistance (*R*_s_), as shown in Figure 9. The equivalent circuit of a solar cell allows us to infer through Equation (1) a relation between the current and the voltage of the cell (*I*_CELL_, *V*_CELL_), as a function of *I*_ph_, the photocurrent density, *I*_0_, the dark current (reverse saturation current of the diode), *R*_s_ and *R*_sh_, the series and shunt resistances of the cell [63]. In Equation (1) *T* is the absolute cell temperature, *m* the diode ideality factor, *k*_B_, the Boltzmann constant and *q*_e_ the electron charge.

(1) $$I_{\mathrm{CELL}}=I_{\mathrm{ph}}-I_{0}\left(e^{\frac{-q_{e}\left(V_{\mathrm{CELL}}+I_{\mathrm{CELL}}R_{S}\right)}{mk_{B}T}}-1\right)+\frac{V_{\mathrm{CELL}}+I_{\mathrm{CELL}}R_{s}}{R_{\mathrm{sh}}}$$

For an ideal cell, *R*_sh_ is infinite, whereas *R*_s_ is zero. In the *I-V* plot of the ideal cell, near the short-circuit point, the curve was roughly horizontal, indicating that the shunt resistance was high. Near the other important point, the open-circuit limit, the *I-V* curve was close to vertical, meaning that the series resistance was low.

When the shunt resistance is very high but the series resistance cannot be neglected, the third term in Equation (1) becomes negligible. In this case, close to the short-circuit point the curve is roughly horizontal, but near the open-circuit point the *I-V* curve is not vertical, suggesting significant series resistive losses in the cell. Therefore, the shape of the *I-V* curves in Figure 8 points to significant series losses.

Examining Table 1 and Table 3 we note that, for the buffer layer deposited in oxygen, *J*_sc_ decreased from 8.136 mA/cm^2^ to 7.907 mA/cm^2^, while for the buffer layer deposited in argon *J*_sc_ decreased from 7.537 mA/cm^2^ to 7.512 mA/cm^2^. Increasing the buffer layer thickness led to a decrease of *J*_sc_, as the wider films have a lower transmission (seen in Figure 7), such that less photons reach the active mesoporous film, and fewer photoelectrons are generated. Moreover, the diffusion of the photoelectrons towards the FTO was hindered by thicker buffer layers.

Finally, the highest value of efficiency (2.83%) was reached for TO10, suggesting that the lower the pressure for the deposition of the buffer layer, the higher the performance of the final device is.

To understand these results one needs to examine the basic processes that occur in the DSSCs [64]. Processes such as charge injection into TiO_2_, charge diffusion to FTO and dye regeneration are desirable. In contrast, the nonradiative decay, the back transfer to dye and the charge interception by electrolyte are detrimental processes [64].

Our results suggest that the higher *J*_sc_ for the devices with the buffer layer is likely due to an increased charge transfer from the TiO_2_ mesoporous layer to the FTO, through the buffer layer (possibly caused by the superior conductivity of the brush-like nanobar structures shown in Figure 1). Another reason may be the decrease in charge loss through charge interception by the electrolyte (which does not get in contact with the fluorine-doped transparent oxide).

The lower fill factor indicates that the introduction of a buffer layer increases the series resistance, *R*_s_. The large *R*_s_ was reflected in the deviation from verticality of the current-voltage curve near the open-circuit point (Figure 8). We suggest that a larger buffer layer thickness, meaning an increased length over which the charges should diffuse, hinders the electron flow and facilitates the charge interception.

We conclude that although the photovoltaic conversion efficiencies obtained in our study were relatively low compared to the existing records, the fact that the buffer layer more than doubles the performance should be emphasized, as it shows promise. It strengthens the opinion that PLD has much to offer in photovoltaics [46,65,66], making it a relevant method for improving the fabrication of TiO_2_-based DSSCs with superior conversion efficiency.

## 5. Conclusions

Our study aimed to increase DSSC performance by interposing an intermediary TiO_2_ buffer layer deposited by PLD. The hint was that an improved contact between the FTO and the mesoporous TiO_2_ film would minimize the resistive losses and increase the short circuit current density by preventing the electrolyte from getting in contact with the FTO.

The buffer layer was deposited in either ambient oxygen or argon at different pressures. The PLD films were comparatively analyzed by transmittance measurements and SEM, TEM and XRD investigations.

Top-view and cross-section SEM micrographs of the TO and TAR films revealed round particulates with homogeneous shape and size dimension. The low-pressure samples displayed thinner PLD layers, whereas the high pressure ones had longer TiO_2_ nanobars. The XRD analyses of TAR10, TO10 and TO50 samples evidenced a monophasic structure, exhibiting maxima only of TiO_2_-anatase. In the case of TAR50 samples no TiO_2_ peaks were observed, thus suggesting an amorphous structure.

TEM images with SAED patterns of the films have confirmed the anatase phase of TiO_2_. Moreover, the anatase phase has also been identified from the 0.359 nm lattice fringes, visible in the HRTEM images. TEM studies of TAR50 samples clearly indicated the presence of the rutile phase but also suggested a mixture of crystalline and amorphous regions.

Electro-optical measurements carried out, under standard AM 1.5G conditions, have shown that the insertion of a buffer layer at the interface of FTO/TiO_2_ led to photovoltaic conversion efficiencies more than two times larger than those of the standard cells. The best performance was recorded for buffer layers deposited in 10 Pa O_2_, which are characterized by an open circuit voltage, *V*_oc_ as high as 608 mV and a short circuit current, *I*_sc_ of 6.39 mA.

The processes that take place inside the device were discussed and the role of the brush-like buffer layer in the performance increase was emphasized. The higher *I*_sc_ for the devices with the buffer layer is likely associated to an increased charge transfer from the TiO_2_ mesoporous layer to the FTO, through the buffer layer as well as to a decrease in charge loss due to charge interception by the electrolyte. The lower *FF* indicates that the introduction of a buffer layer increases the series resistance, *R*_s_, due to an increased length over which the charges have to diffuse, hindering the electron flow and facilitating the charge interception.

The goal of the present study was to find the PLD parameters that optimize the performance of the DSSCs. The fact that the buffer layer more than doubled the performance is to be emphasized, as it shows promise. Despite the relatively low efficiency obtained, which indicates that the fabrication technology was not yet optimized, the increase reported here was significant and exceeds the enhancements of 15–80% stated for other techniques as well as that of up to 42% for the same method. Our results strengthen the opinion that PLD has much to offer in photovoltaics, making it an interesting method for obtaining oxide semiconductor nanostructures for DSSCs.

Finally, it should be noted that the long-term device performance is critical for applications. It would clearly verify the role of the intermediate TiO_2_ buffer layer on the good mechanical contact, protection of the electrodes against the dye solution action, oxidation at operating temperatures, as well as the reduced recombination of electrons at the electrode/electrolyte interface. Such studies, which require a long time, are underway and will be subject of a subsequent report.

## Figures and Tables

**Table 1:** Thickness of TiO_2_ mesoporous coatings and of TiO_2_ buffer layers deposited by PLD, on a SnO_2_:F conductive film of 0.41 µm.

Film Type	TiO_2_ Mesoporous Film	TO10 Buffer Layer	TO50 Buffer Layer	TAR10 Buffer Layer	TAR50 Buffer Layer
Thickness (µm)	~29.89	~0.65	~2.95	~0.33	~1.24

**Table 2:** Buffer layers: Structural and morphological features.

TiO_2_ Layer Type	XRD (101) Crystalline Coherent Length (nm)	TEM Mean Grain Size (nm)
TO10	~21.5	12.6
TO50	~28.1	12.0
TAR10	~40.8	16.9
TAR50	n/a	13.3
Mesoporous film	~19.0	16.4

**Table 3:** Electro-optical parameters: Short circuit current (*I*_sc_), open circuit voltage (*V*_oc_), short circuit current density (*J*_sc_), maximum power (*P*_max_), fill factor (*FF*) and photovoltaic conversion efficiency (*η*) of typical dye-sensitized solar cells (DSSCs) measured under standard illumination conditions (Figure 9).

Sample	*I*_sc_ (mA)	*V*_oc_ (mV)	*J*_sc_ (mA/cm^2^)	*P*_max_ (μW)	*FF*	*η* (%)
TO10	6.39	608	8.136	2228	0.57	2.84
TO50	5.92	604	7.537	2015	0.56	2.57
TAR10	6.21	594	7.907	2041	0.55	2.60
TAR50	5.90	609	7.512	1978	0.55	2.52
No buffer	2.48	590	3.166	968	0.66	1.23

## Supplementary Materials

The following are available online at https://www.mdpi.com/2079-4991/9/5/746/s1, Figure S1: Selected area electron diffraction (SAED) patterns for the mesoporous TiO_2_ film (a), and the buffer layers: TO10 (b), TO50 (c), TAR10 (d), and TAR50 (e).
