# Supplementary material for: Pulsed Laser Fabrication of TiO2 Buffer Layers for Dye Sensitized Solar Cells
Source: Nanomaterials (Basel). 2019 May 15;9(5):746. doi: 10.3390/nano9050746 (PMC6566938; doi:10.3390/nano9050746)
Supplement: Supplementary file 1 [file nanomaterials-09-00746-s001.pdf]

nanomaterials

Supplementary Materials

Pulsed laser fabrication of TiO_2 buffer layers for dye sensitized solar cells

Jeanina Lungu[1], Gabriel Socol[2], George E. Stan[3], Nicolaie Ştefan[2], Cătălin Luculescu[2], Adrian Georgescu[1], Gianina Popescu-Pelin[2], Gabriel Prodan[1], Mihai A. Gîrţu[1,]* and Ion N. Mihăilescu[2]*

Supplementary Materials:

1. Figure S1: Selected area electron diffraction (SAED) patterns for the mesoporous TiO_2 film (a), and the buffer layers: TO10 (b), TO50 (c), TAR10 (d), and TAR50 (e).

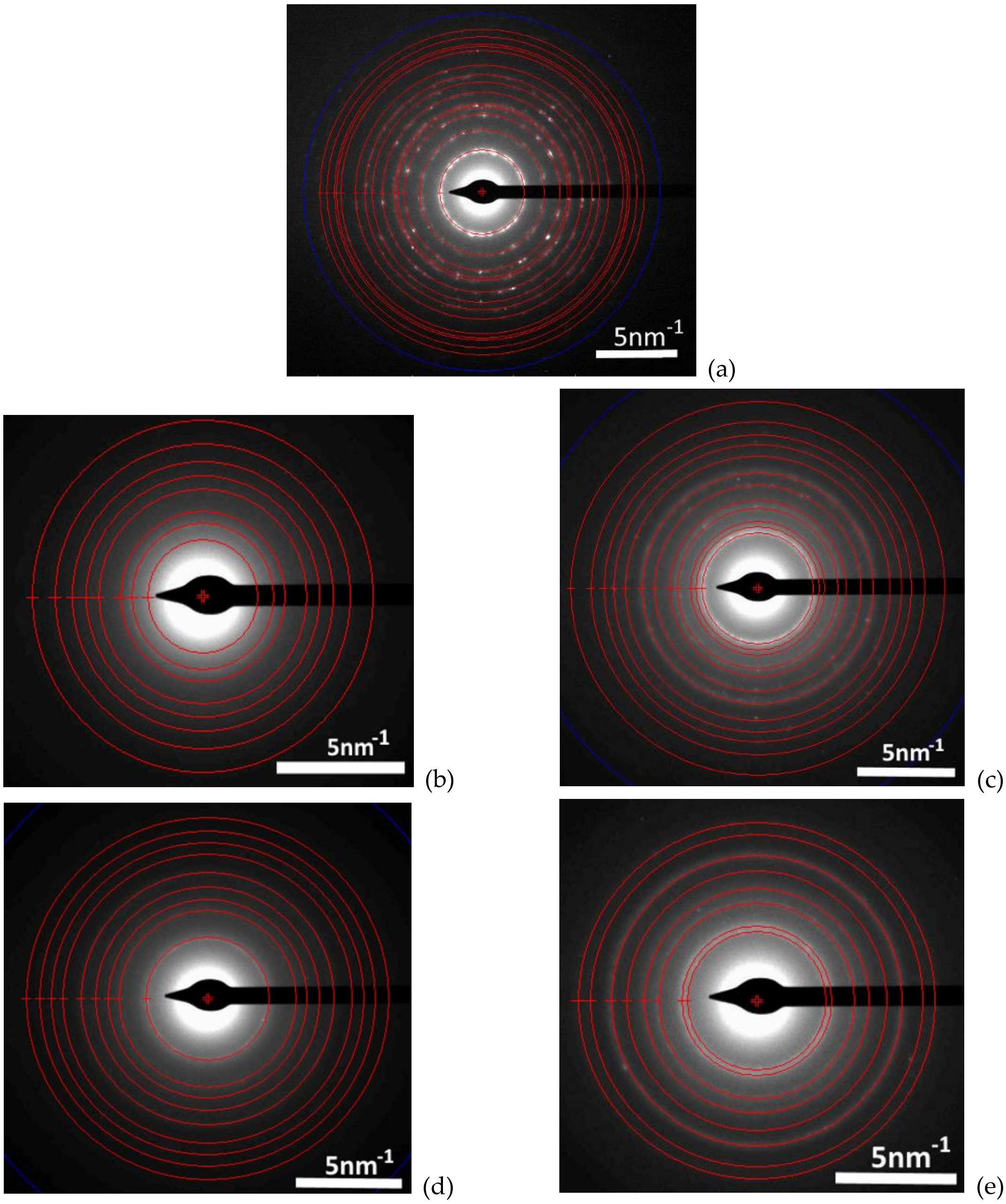

Figure S1. Selected area electron diffraction (SAED) patterns for the mesoporous TiO_2 film (a), and the buffer layers: TO10 (b), TO50 (c), TAR10 (d), and TAR50 (e).
